# Long-term low-dose exposure of permethrin induces liver and kidney damage in rats

**DOI:** 10.1186/s40360-022-00586-2

**Published:** 2022-07-08

**Authors:** Ying-Jian Sun, Yu-Jie Liang, Lin Yang, Ding-Xin Long, Hui-Ping Wang, Yi-Jun Wu

**Affiliations:** 1 Laboratory of Molecular Toxicology, Institute of Zoology, Chinese Academy of Sciences, 1-5 Beichenxilu Road, Beijing, 100101 P. R. China; 2 Department of Veterinary Medicine and Animal Science, Beijing University of Agriculture, Beijing, 102206 P. R. China; 3 Shenzhen Kangning Hospital, Shenzhen, 518020 Guangdong China

**Keywords:** Insecticide, Pyrethroid, Long-term low-dose exposure, Histopathology, Hepatotoxicity, Nephrotoxicity

## Abstract

**Background:**

Permethrin is one of the pyrethroid insecticides, which is widely used in agriculture and public health. Although acute toxicity of the insecticide has been studied, the chronic toxicity upon the long-term exposure has not been clear yet. The purpose of the current study is to investigate the organ toxicities of permethrin following its long-term low-dose exposure.

**Methods:**

Male Wistar rats were daily administrated orally with permethrin (75 mg/kg body weight/day, gavage) for 90 days, and then the samples of biofluids (blood and urine) and organs including liver and kidney were collected. The serum and urine samples were measured by biochemical assay and the tissues of kidney and liver were examined and analyzed by histopathological method.

**Results:**

The results showed that no change was found in serum and urine biochemical parameters for the toxicity; however, significant changes including hyperchromatic nuclei swollen in the hepatic parenchymal cells and the swelling proximal tubules in the kidneys were observed in the tissue structures of liver and kidneys in the histopathological sections.

**Conclusion:**

These results indicate that low-dose long-term exposure of permethrin can cause chronic toxicity with slight liver and kidney damage.

## Introduction

Synthetic pyrethroids are a group of insecticides widely used in agriculture and public health, which comprise over one-third of all insecticides in the world [1]. Pyrethroid insecticides demonstrate a selective toxicity toward insects. They are mainly used for mosquito eradication and pests control [1–5]. Pyrethroid insecticides are considered to be one of the safest pesticides available, but the potential toxicity risk of the insecticides following long-term exposure has been concerned and debated [6, 7]. Recent studies have suggested that the pyrethroid pesticides are not entirely safe to mammals’ health [8, 9]. Permethrin is one of the most used pyrethroid insecticides, which is characterized by low toxicity to vertebrates including mammals but high toxic against target insects. It is known that permethrin produces toxicity through targeting the important site of sodium channels to induce a decrease of the activity of the channels in nervous system [10–13]. The long-term wide use of this insecticide produces an increasing concern for the health of humans [14].

Some investigations have been carried out on the high-dose short-term toxicity of permethrin [15, 16]. In reality, however, the actual exposure to the insecticide is usually at low-level and for long-term [17, 18]. The majority of previous investigations on permethrin have focused on the target toxicities, for example, the neurotoxicity [4, 19–21]; however, there has been minimal study conducted on the organ toxicity of the insecticide to mammals after low-dose and long-term exposure. In this investigation, we studied the effect of permethrin on the liver and kidney functions in rats following 90-day low-dose oral exposure.

## Materials and methods

### Chemicals

Permethrin (40 60 cis trans isomer ratio, 95% purity) was purchased from Ronch Chemical Co. (Nanjing, China). Hematoxylin, eosin, and other chemicals were obtained from Sigma Chemical Co. (St. Louis, MO, USA).

### Animals and treatment

Ten male Wistar rats (200 ± 20 g) were obtained from Weitong Lihua Laboratory Animal Technology Company (Beijing, China) and were housed individually in cages. All animals were acclimatized for at least 1 week prior to the study [22]. During the experiment, the animal rooms were maintained at 22 ± 2 °C temperature and 50–60% humidity and a light/dark cycle of 12 h. Animals had free access to water and the diet. The rats were randomly divided into 2 groups (control and permethrin treatment groups) with 5 animals in each group. Previous studies showed that acute oral half-lethal dose (LD_50_) of permethrin was 1500 mg/kg for male rats [23]. In this study we chose the value of 1/20 LD_50_ as the dose (75 mg/kg body weight/day) for the pesticide treatment rats.

The pesticide was dissolved in corn oil (it is difficult to be dissolved in water) and then orally administered daily via gavage to rats (1 ml/kg body weight). Rats were given permethrin daily (once a day) for 90 days. The rats in control group received daily an equivalent dose of corn oil. The body weight of each rat was recorded daily, and symptoms and conditions of the rats were monitored daily throughout the experiment.

All animal procedures were performed in accordance with current China legislation and approved by the Animal and Medical Ethics Committee from Institute of Zoology, Chinese Academy of Sciences.

### Samples collection and preparation

The sample collection and preparation were carried out in the similar way as our earlier report [24]. Briefly, after the last administration, 24-hour urine samples of each rat were collected into an ice-cold vessel containing 0.1 ml of 1% sodium azide to prevent bacterial contamination and the samples were stored at − 80 °C prior to biochemical analysis.

All rats were anesthetized with pentobarbital sodium and decapitated twenty-four hours after the last administration. Blood samples were collected and then centrifuged to obtain the serum for biochemical assays. Weights of organs were immediately measured after they were isolated from the body.

### Histopathology

For histopathological analysis, the kidney and liver tissues of the rats were dissected immediately at the end of the 90-day exposure to the pesticide, and then were fixed in a solution of 10% formalin. After fixation, the specimens were dehydrated with 80, 95, 100% gradient alcohol, and then soaked in melted wax, and finally embedded in paraffin [25]. After that, the paraffin blocks were cut into 4-μm-thickness sections using a microtome (Microm HM 340E, Thermo Fisher Scientific, USA), then were stained with hematoxylin and eosin. The histological changes in the kidney and liver tissue sections were examined in a microscope (Olympus, Tokyo, Japan) and evaluated by a pathologist blinded to the different treatment groups.

### Serum and urine biochemistry

All biochemical parameters of serum and urine samples were analyzed on an Autolab-PM4000 automatic analyzer (AMS Co.), according to the standard spectrophotometric methods. Values of the biochemical parameters were expressed as the mean ± SD.

### Statistical analysis

Student’s *t*-test was used to assess the statistical significance of differences in measured parameters between the two groups. *P* < 0.05 was considered statistically significant. SPSS 18.0 software (SPSS, Inc., Chicago, MI, USA) was employed for the statistical evaluation.

## Results

### Effects of permethrin on body weight and organ weight

During the 90-day experimental period, no obvious toxic sign was observed in the control rats and the permethrin-treated rats. And no animal was found dead during the whole experimental period of the study.

The weights of liver, kidneys, and other organs in permethrin-treated rats were not changed significantly after the 90-day exposure compared with those in control rats (Table 1), although the body weight of the treated rats decreased. The slight decrease (about 12%) of the body weight in permethrin-treated rats was perhaps due to the less feed intake.

**Table 1:** Effect of permethrin exposure on the relative organ weights of the rats

Parameters	Control	Permethrin
Terminal body weight (g)	479.7 ± 13.3	421.4 ± 13.7^*^
Brain (g%)	0.437 ± 0.027	0.479 ± 0.036
Heart (g%)	0.275 ± 0.010	0.293 ± 0.010
Liver (g%)	2.94 ± 0.17	2.76 ± 0.27
Spleen (g%)	0.202 ± 0.021	0.205 ± 0.018
Kidneys (g%)	0.667 ± 0.073	0.705 ± 0.025
Testicles (g%)	0.861 ± 0.062	0.875 ± 0.076
Adrenals (mg%)	16.2 ± 3.7	18.2 ± 2.4

Rats were given permethrin (75 mg/kg body weight/day, gavage) daily for 90 consecutive days. The rats in control group received daily an equivalent dose of corn oil (1 ml/kg body weight/day). The body weight of each rat was recorded daily. All rats were anesthetized with pentobarbital sodium and decapitated twenty-four hours after the last administration. Weights of organs were immediately measured after they were isolated from the body. Data are presented as mean ± SD (*n* = 5). ^*^P < 0.05, compared with the control

### Effect of permethrin on the clinical biochemistry

The changes of the biochemical parameters in serum and urine of the rats are shown in Table 2. We found that there was no significant change in the biochemical parameters of the serum and urine samples. These results indicate that the typical serum and urine biochemistry did not display obvious toxicity after 90-day exposure of permethrin.

**Table 2:** Biochemical analysis of serum and urine from the rats exposed to permethrin

Samples	Parameters	Control	Permethrin
Serum	SALP (U/L)	53.00 ± 7.82	66.80 ± 20.06
	SGOT (U/L)	109.75 ± 19.62	99.80 ± 10.39
	SGPT (U/L)	46.25 ± 7.65	44.20 ± 5.01
Urine	UUM (mM)	59.72 ± 5.81	46.73 ± 10.65
	UCRE (mM)	1902.3 ± 260.8	1882.3 ± 322.2

Rats were given permethrin (75 mg/kg body weight/day, gavage) daily for 90 consecutive days. The rats in control group received daily an equivalent dose of corn oil (1 ml/kg body weight/day). After the last administration, 24-hour urine samples of each rat were collected. And then all rats were anesthetized with pentobarbital sodium and decapitated. Blood samples were collected and then centrifuged to obtain the serum for biochemical assays. The biochemical parameters in the samples were analyzed by an automatic analyzer. Data are presented as mean ± SD (*n* = 5).

*Abbreviations*: *SALP* serum alkaline phosphatase, *SGOT* serum glutamic-oxaloacetic transaminase, *SGPT* serum glutamic pyruvic transaminase, *UUN* urine urea nitrogen, *UCRE* urine creatine

### Effect of permethrin on histopathology of liver and kidneys

**Fig. 1:**
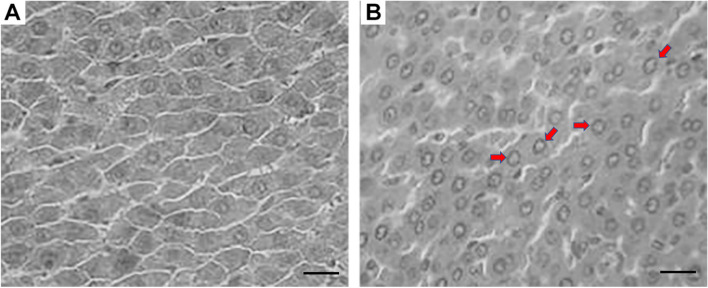
Effect of permethrin on the liver of rats. The representative sections of liver tissue of the rats were examined under a microscope. Compared with the control rats showing normal structure of hepatocytes (**A**), the rats exposed to permethrin (75 mg/kg body weight/day) for 90 consecutive days exhibited prominent swollen hyperchromatic nuclei (red arrows) (**B**). Scale bar: 20 μm

**Fig. 2:**
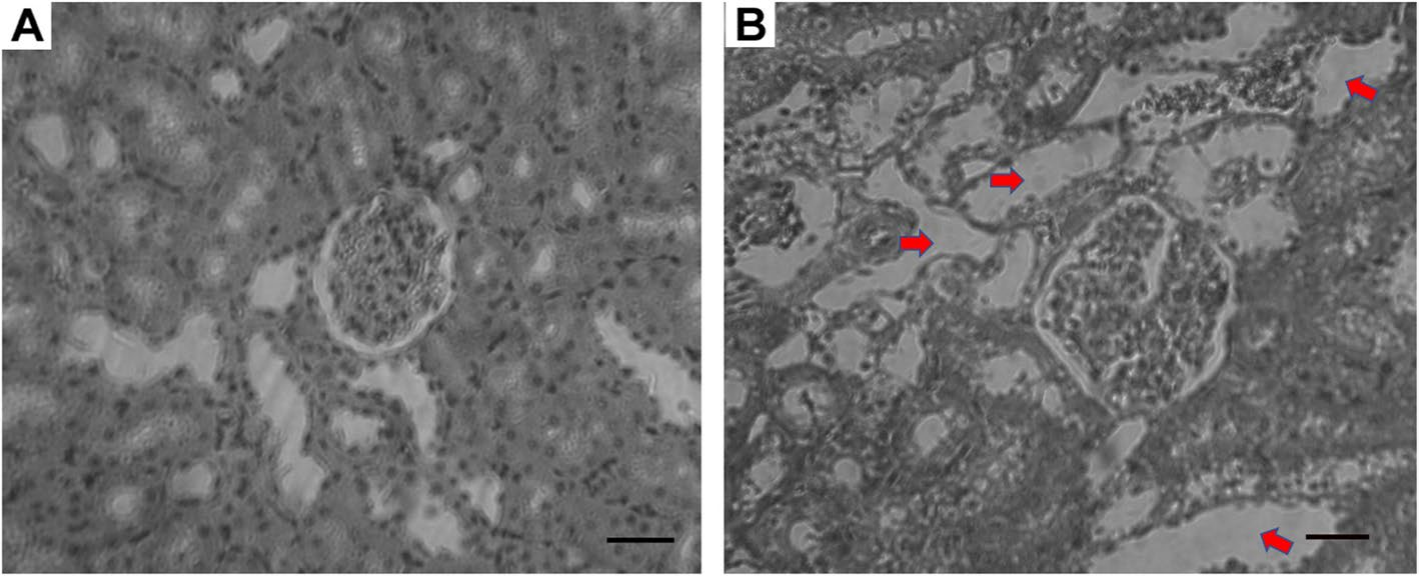
Photomicrographs of representative sections of the kidney of the treated rats. No obvious abnormal structure was found in the kidneys from the control rats (**A**); however, the swelling proximal tubules (red arrows) were observed in kidneys of the rats exposed to the permethrin (75 mg/kg body weight/day) for 90 consecutive days (**B**). Scale bar: 20 μm

Photomicrographs of the liver and kidney tissues are shown in Figs. 1 and 2, respectively. The microscope examination of liver sections displayed that the hyperchromatic nuclei were swollen in the hepatic parenchymal cells from the rats exposed to permethrin (Fig. 1), suggesting that permethrin induced slight liver damage after 90-day exposure at the dose of 75 mg/kg body weight/day. In the kidney sections, the swelling proximal tubules were observed (Fig. 2), which suggests that the long-term low-dose exposure of permethrin could also induce the kidney damage.

## Discussion

Permethrin which is used as a pyrethroid insecticide can affect sodium channels in the insect nervous system. The symptoms of acute toxicity for human and other vertebrates is vomiting, dyspnea, cough and bronchospasm, and skin effects [26]. There is a lot of evidence that exposure to permethrin can cause reproductive toxicity, and endocrine disrupting [27]. However, the long-term health implications of exposure to pyrethroids, especially the effects of the insecticide on important organs and tissues, are still unclear.

In this study, we used Wister rat to study the organ toxicity of pesticide permethrin. Rat has been using as a test animal for the study of hepatotoxicity and nephrotoxicity of chemicals since rat is in similar toxicological symptom and tissue structure change to human. In this investigation, we determined the changes of body weight and organ weight, biochemical parameters of serum and urine, and the histopathological changes of the kidneys and liver from the rats following 90-day exposure to low-dose permethrin. The dose used (75 mg/kg body weight/day, 1/20 LD_50_) was comparable to the reported chronic no-observed-adverse-effect level (NOAEL) in rats [28]. It was inferred from the results, combined with the data from the previous reports that pesticide permethrin exposure even at low dose could affect the organs functions, although no obvious pathological change was found in liver and kidneys of the rats exposed to permethrin for a relatively shorter time [24, 29, 30]. In our study, some of the nuclei are massively enlarged, abnormally stained in the sections of liver from the permethrin-treated rats. The morphological changes in the sections of kidney revealed the swelling of the proximal tubules, suggesting the histology of megakaryocytic interstitial nephritis. The results in this study suggested that permethrin could cause the damage of liver and kidneys even at low dose after long-term exposure and even at that time no any apparent toxic symptom or abnormal change of the biochemical parameters could be observed.

## Data Availability

The datasets used and analyzed during the current study are available from the corresponding author on reasonable request. All data generated or analyzed during this study are included in this published article.
